# Metronomic Cordycepin Therapy Prolongs Survival of Oral Cancer-Bearing Mice and Inhibits Epithelial-Mesenchymal Transition

**DOI:** 10.3390/molecules22040629

**Published:** 2017-04-13

**Authors:** Nai-Wen Su, Shu-Hua Wu, Chih-Wen Chi, Chung-Ji Liu, Tung-Hu Tsai, Yu-Jen Chen

**Affiliations:** 1 Division of Medical Oncology and Hematology, Department of Internal Medicine, Mackay Memorial Hospital, Taipei 11094, Taiwan; medicine_su@hotmail.com; 2 Institute of Traditional Medicine, School of Medicine, National Yang-Ming University, Taipei 11221, Taiwan; 3 Department of Medical Research, Mackay Memorial Hospital, Taipei 25160, Taiwan; lazycat2233@yahoo.com.tw (S.-H.W.); cwchid48906003@gmail.com (C.-W.C.); 4 Department of Oral and Maxillofacial Surgery, Mackay Memorial Hospital, Taipei 11094, Taiwan; cjliu3229@gmail.com; 5 Institute of Oral Biology, School of Dentistry, National Yang-Ming University, Taipei 11221, Taiwan; 6 Department of Chemical Engineering, National United University, Miaoli 36063, Taiwan; 7 Department of Radiation Oncology, Mackay Memorial Hospital, Taipei 25160, Taiwan; 8 Research Center for Chinese Medicine and Acupuncture, China Medical University, Taichung 404, Taiwan

**Keywords:** cordycepin, epithelial-mesenchymal transition, oral squamous cell carcinoma metronomic therapy

## Abstract

Cordycepin (3′-deoxyadenosine) is a natural compound abundantly found in *Cordyceps sinesis* in natural and fermented sources. In this study, we examined the effects of cordycepin in a human oral squamous cell carcinoma (OSCC) xenograft model. Cordycepin was administered in a regular, low-dose and prolonged schedule metronomic therapy. Two doses of cordycepin (25 mg/kg, 50 mg/kg) were administrated five days a week for eight consecutive weeks. The tumor volumes were reduced and survival time was significantly prolonged from 30.3 ± 0.9 days (control group) to 56 days (50 mg/kg group, the day of tumor-bearing mice were sacrificed for welfare consideration). The weights of mice did not change and liver, renal, and hematologic functions were not compromised. Cordycepin inhibited the OSCC cell viability in vitro (IC_50_ 122.4–125.2 μM). Furthermore, morphological characteristics of apoptosis, increased caspase-3 activity and G2/M cell cycle arrest were observed. In wound healing assay, cordycepin restrained the OSCC cell migration. Cordycepin upregulated E-cadherin and downregulated N-cadherin protein expression, implying inhibition of epithelial-mesenchymal transition (EMT). The immunohistochemical staining of xenograft tumor with E-cadherin and vimentin validated in vitro results. In conclusion, metronomic cordycepin therapy showed effective tumor control, prolonged survival and low toxicities. Cytotoxicity against cancer cells with apoptotic features and EMT inhibition were observed.

## 1. Introduction

Head and neck squamous cell carcinoma (HNSCC) is the sixth most common cancer worldwide [1]. In some areas, the incidence rate is outnumbered by HNSCC of the oral cavity [2]. The carcinogenic factors include tobacco, alcohol consumption and most importantly, betal nuts chewing in the prevalent areas in Asia [3]. More than 50% of patients with HNSCC present at the locally advanced stage [4]. The multidisciplinary treatment strategy is the cornerstone of the locally advanced HNSCC, including radical surgery, radiotherapy and chemotherapy [5]. However, the long-term disease-free survival among these patients is only 40–50% and the combination treatment results in significant acute and late toxicities [6]. The prognosis for recurrent or refractory disease status is dismal. The median survival was about 10 months with combined anti-epidermal growth factor receptor (EGFR) monoclonal antibody and platinum-based chemotherapy [7].

In the field of systemic cancer treatment, metronomic chemotherapy is characterized by close, regular administration of chemotherapeutic drugs at relative low, minimally toxic doses for a long period with no extended drug-free break [8]. Apart from direct antitumor effects, metronomic chemotherapy was shown to have other mechanisms, such as anti-angiogenesis and reverse of immunosuppressive microenvironment [9,10]. Due to better chemotherapeutic tolerability, metronomic treatment strategy has been tested in clinical practice [11].

With rapid advances in molecular biology research, researchers found that cancer cells possess 10 characteristics that enhance their malignant behavior [12]. One of these is epithelial-mesenchymal transition (EMT), which cancer cells adopt to increase the ability of local invasion and distant metastasis [12]. In several cancer types, cells harboring EMT-related molecular markers imparted poor prognosis [13,14]. Similar outcomes were demonstrated in HNSCC-related research [15]. Moreover, EMT results in a higher possibility of recurrence and metastasis in HNSCC [16]. Thus, EMT inhibition is a reasonable target in HNSCC treatment to prevent cancer recurrence and distant spread. This is an important research focus in cancer treatment [17].

**Figure 1:**
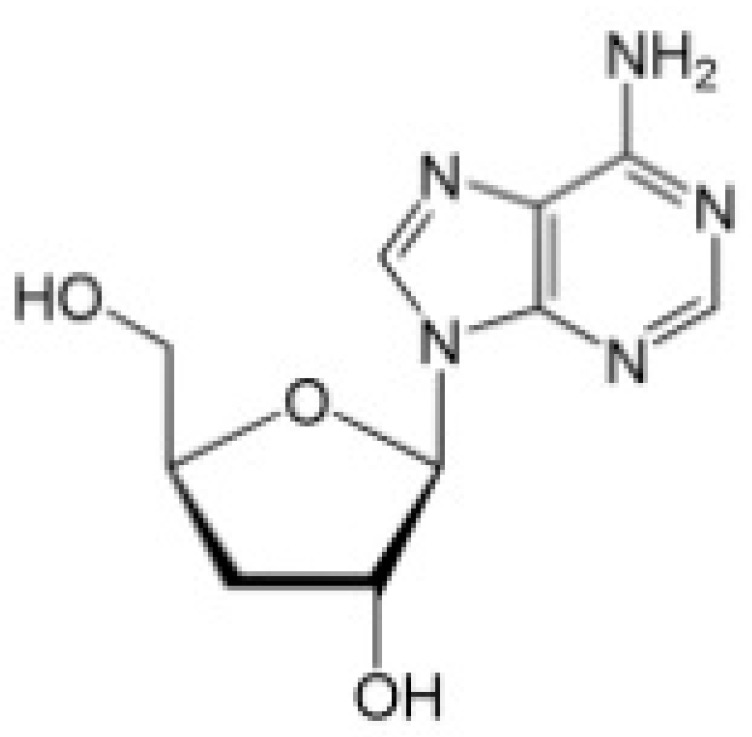
Chemical structure of Cordycepin isolated from *Codyceps sinensis* and *Cordyceps militaris*.

*Cordyceps sinensis* is one of the most famous, precious, and commonly used Chinese medicinal herbs. It is well-known as “winter worm, summer grass” as it is a parasitic habitant on the larvae or pupae of insects [18]. Cordycepin (3′-deoxyadenosine) is an adenosine analogue and one of the major compounds isolated from *C. sinensis* (Figure 1) [19]. Various biological activities have been demonstrated, including immunomodulation, neuroprotective, steroidogenesis, lipid-lowering, osteogenesis, and antimicrobial [20,21]. Importantly, cordycepin has anticancer potential, which may be exerted through the inhibition of RNA/DNA biosynthesis, induction of apoptosis, anti-angiogenesis, and immunomodulation [22]. Cordycepin can inhibit proliferation and invasion in several cancer types [23,24]. While the metastatic potential of cancer cells often results in the disease being incurable, cordycepin can reverse the process in vitro and in vivo [25,26].

In our study, we tested the anticancer effects of cordycepin on two oral squamous cell carcinoma (OSCC) cell lines, including its cytotoxicity and antimigration ability. We also demonstrated that cordycepin could inhibit EMT in vitro and OSCC tumor growth in xenograft tumor-bearing mice model with a prolonged metronomic administration schedule.

## 2. Results

### 2.1. Cordycepin Inhibits Xenograft Tumor Growth and Prolongs Survival

**Figure 2:**
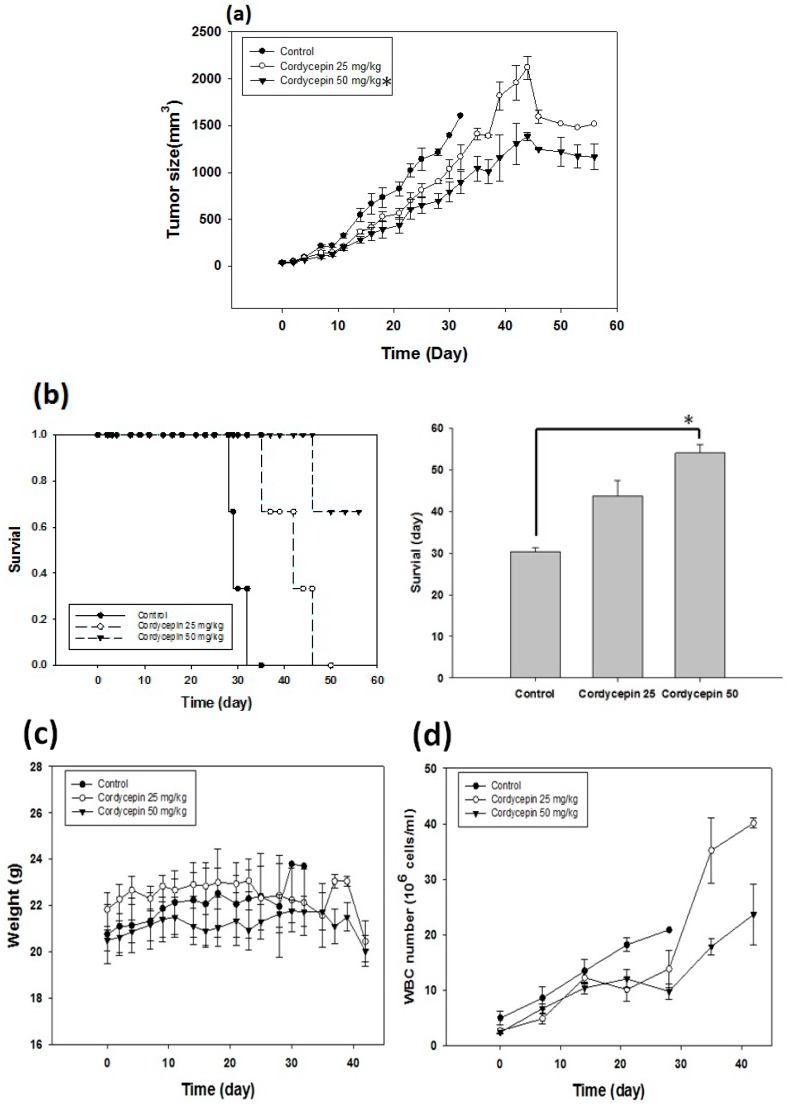

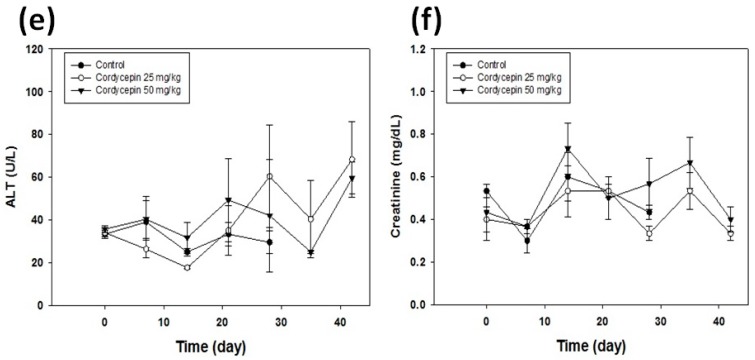
Cordycepin treatment inhibited tumor growth, prolonged survival time and maintained organ functions. The tumor-bearing mice received PBS, and 25 mg/kg or 50 mg/kg cordycepin five days a week for eight weeks. (**a**) Cordycepin at 50 mg/kg delayed tumor growth compared with PBS and cordycepin at 25 mg/kg (* *p* < 0.05); (**b**) the survival time was prolonged in 50 mg/kg cordycepin group compared with the other two groups (* *p* < 0.05). The change in weight (**c**) and laboratory parameters including, white blood cell counts (**d**) serum alanine aminotransferase (ALT) levels (**e**), and serum creatinine levels (**f**) were not significantly different among the three groups of tumor-bearing mice.

We treated the tumor-bearing mice with phosphate buffered saline (PBS) and 25 mg/kg or 50 mg/kg cordycepin five days a week for eight weeks. We observed that the tumor growth significantly reduced in the 50 mg/kg cordycepin group compared with 25 mg/kg cordycepin or PBS group (Figure 2a). The survival time was 30.3 ± 0.9 days, 43.7 ± 3.8 days and 54 ± 2.0 days for the PBS, 25 mg/kg and 50 mg/kg cordycepin groups, respectively (Figure 2b). Despite prolonged administration of cordycepin, the weights of mice did not change (Figure 2c). White blood cell counts, liver alanine aminotransferase (ALT) and renal function (serum creatinine) were also not affected under the treatment conditions (Figure 2d–f).

### 2.2. Cordycepin Exerts Growth Inhibition on Cancer Cells

**Figure 3:**
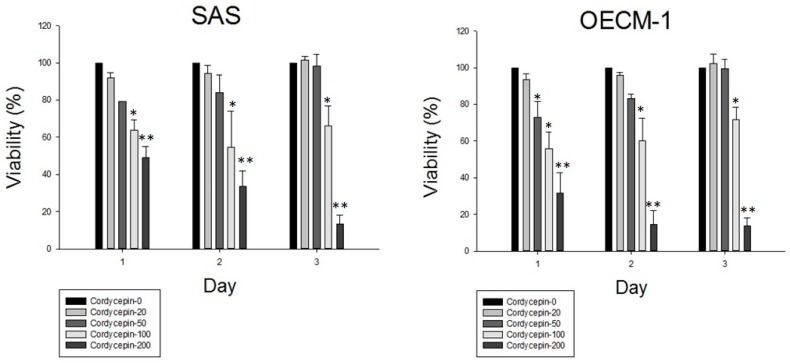

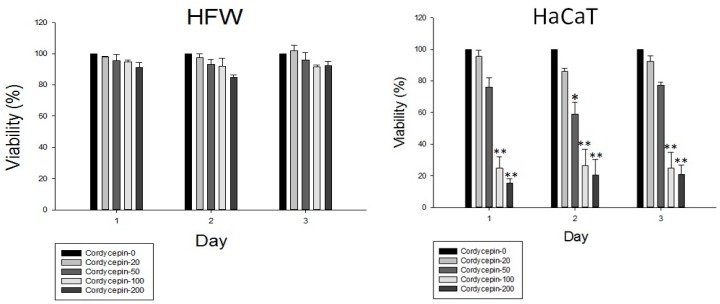
Cordycepin inhibited oral cancer cell proliferation. After 2.5 × 10^4^ cells were seeded into the 24-well plate and grown for 24 h, the cells were treated with 0, 20, 50, 100, 200 μM cordycepin for 24, 48 and 72 h. Then, cells were harvested and MTT assay was performed to evaluate the cell viability. All the experiments were performed in triplicates (* *p* < 0.05, ** *p* <0.01).

Cordycepin inhibited cell growth against two OSCC cancer cells: SAS and OEC-M1. The cancer cell viability decreased in a dose- and time-dependent manner after cordycepin treatment (Figure 3). The concentration at which 50% cell growth inhibition was achieved (IC_50_ with 24 h cordycepin treatment) was 122.4 μM and 125.2 μM for SAS and OEC-M1 cells, respectively. Against the normal counterpart, HFW fibroblast cells, cordycepin showed no cytotoxic effects even at a high concentration of 200 μM. However, cordycepin resulted in cell viability inhibition on another human normal counterpart, keratinocytes HaCaT cells (Figure 3).

### 2.3. Cordycepin Induces G2/M Phase Arrest and Apoptosis of OSCC Cells

**Figure 4:**
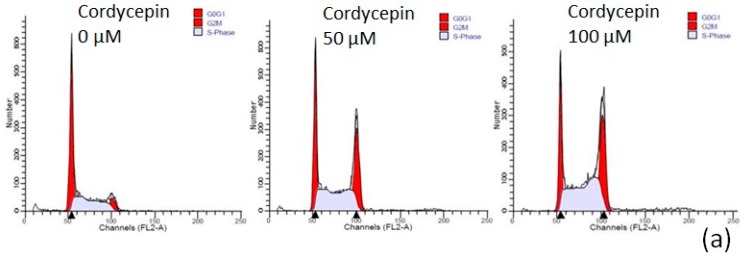

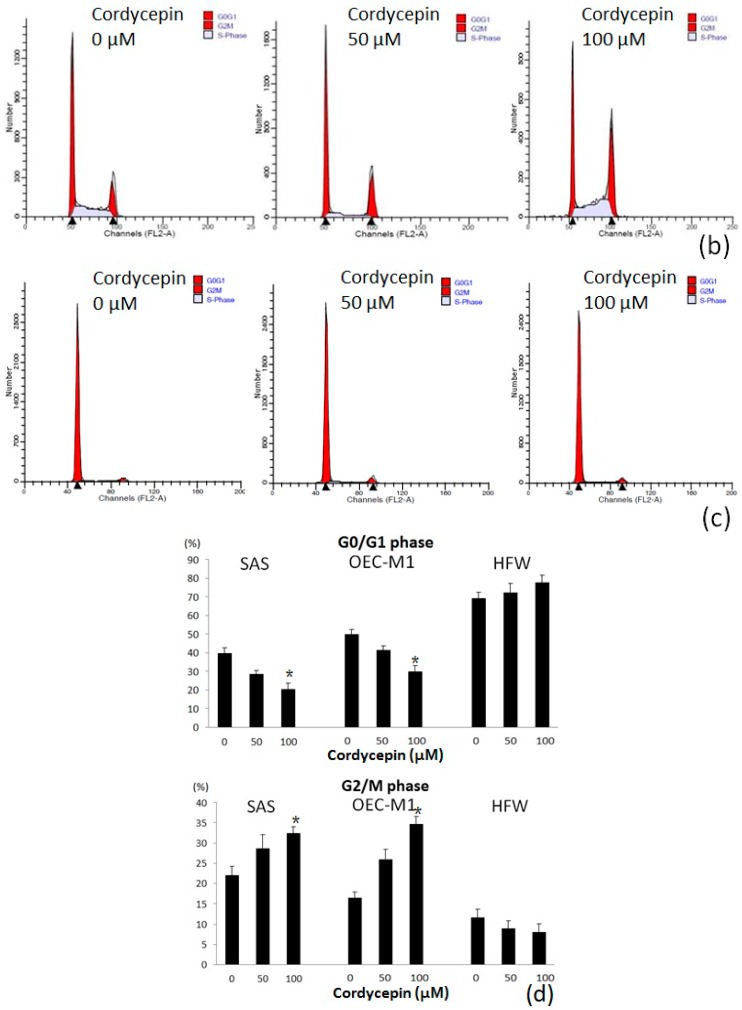
Cordycepin resulted in OSCC cell cycle arrest at G2/M phase. The cells were treated with the indicated concentrations (0, 50 and 100 μM) of cordycepin for 24 h. **a**–**c** shows representative flow cytometry of SAS (**a**), OEC-M1 (**b**), and fibroblast HFW (**c**) cells; (**d**) demonstrated the analysis of cell cycle data with the experiments triplicated (* *p* < 0.05).

**Figure 5:**
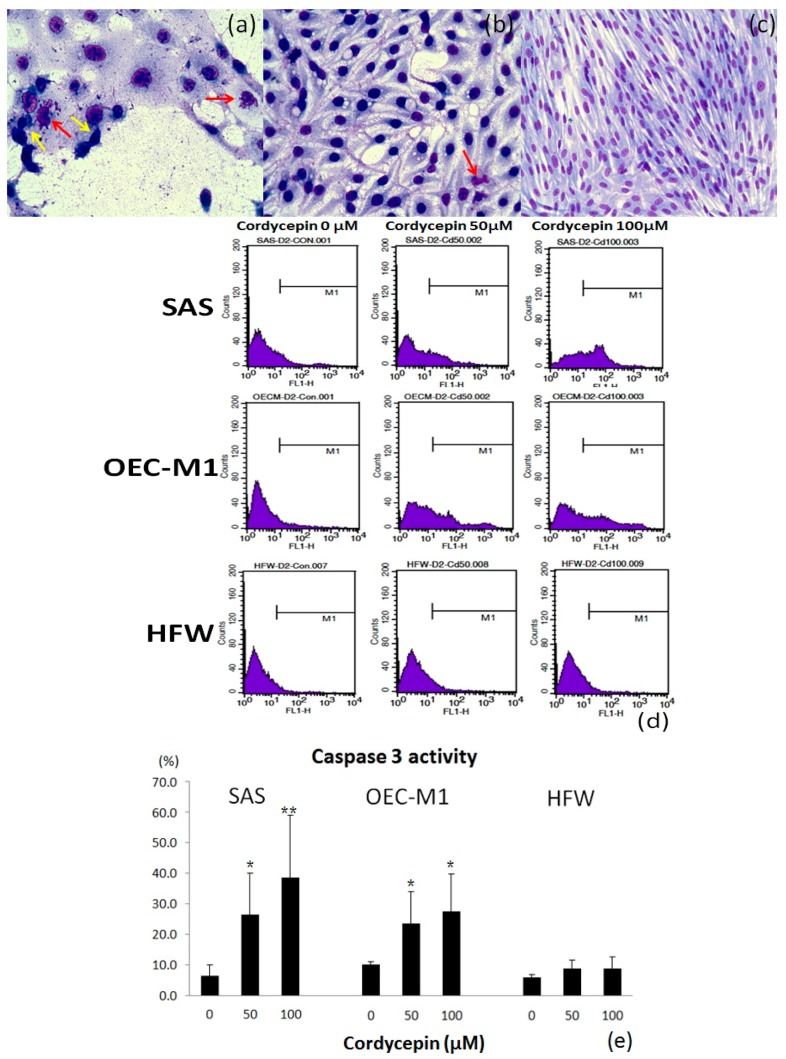
Cordycepin triggered apoptosis in two OSCC cell lines, but not human fibroblasts. The results for Liu’s staining of SAS, OEC-M1 and HFW cells treated with 100 μM cordycepin for 48 h are shown in (**a**–**c**), respectively. The cordycepin-treated OSCC cells showed morphological characteristics of apoptosis, including cell shrinkage (yellow arrow) and apoptotic body formation (red arrow), while the HFW cells were not affected. The caspase-3 activity assay was performed for the three types of cells after 48 h of treatment with placebo, 50 μM and 100 μM cordycepin (**d**). Significantly increased caspase-3 activity was observed on increasing the cordycepin concentration in both the OSCC cells. However, caspase-3 activity did not increase in HFW cells after cordycepin treatment (**e**) (* *p* < 0.05, ** *p* < 0.01).

To investigate the apoptotic effect of cordycepin on the two OSCC cell lines, we performed cell cycle analysis by flow cytometry, Liu’s stain and caspase-3 activity assay. Cordycepin increased G2/M phase arrest on treatment with higher concentration for 24 h (Figure 4a,b). The proportion of SAS cells in G0/G1 phase and G2/M phase after treatment with 0 μM, 50 μM, 100 μM cordycepin was 39.94% ± 2.77% and 20.06% ± 3.19%, 28.56% ± 1.74% and 28.61% ± 3.39%, 20.51% ± 3.17% and 32.47% ± 1.58%, respectively. The proportion of OEC-M1 cells in G0/G1 phase and G2/M phase after treatment with 0 μM, 50 μM, 100 μM cordycepin was 49.96% ± 2.59% and 16.49% ± 1.48%, 41.47% ± 2.37% and 26.02% ± 3.44%, 30.01% ± 3.51% and 34.67% ± 1.88%, respectively. The decrease in G0/G1 phase arrest and increase in G2/M phase arrest of both OSCC cells were significantly with 100 μM cordycepin (*p* < 0.05) comparing with the control group (Figure 4d). However, the cell cycle of human fibroblast HFW cells was not affected by cordycepin treatment (Figure 4c). After Liu’s staining of cordycepin-treated OSCC cells, the cancer cells presented with features of apoptosis, which included cell shrinkage, nuclear condensation and apoptotic body formation (Figure 5a,b). Furthermore, caspase-3 activity assay revealed that caspase-3 was activated in OSCC cells after cordycepin treatment in a dose-dependent manner (Figure 5d,e). In human HFW cells, no changes in Liu’s stain cell morphology (Figure 5c) and caspase-3 activity (Figure 5d,e) were detected after cordycepin treatment. The results suggested that cordycepin resulted in OSCC cell apoptosis and G2/M cell cycle arrest.

### 2.4. Cordycepin Delays Wound Closure by Inhibiting EMT Process

**Figure 6:**
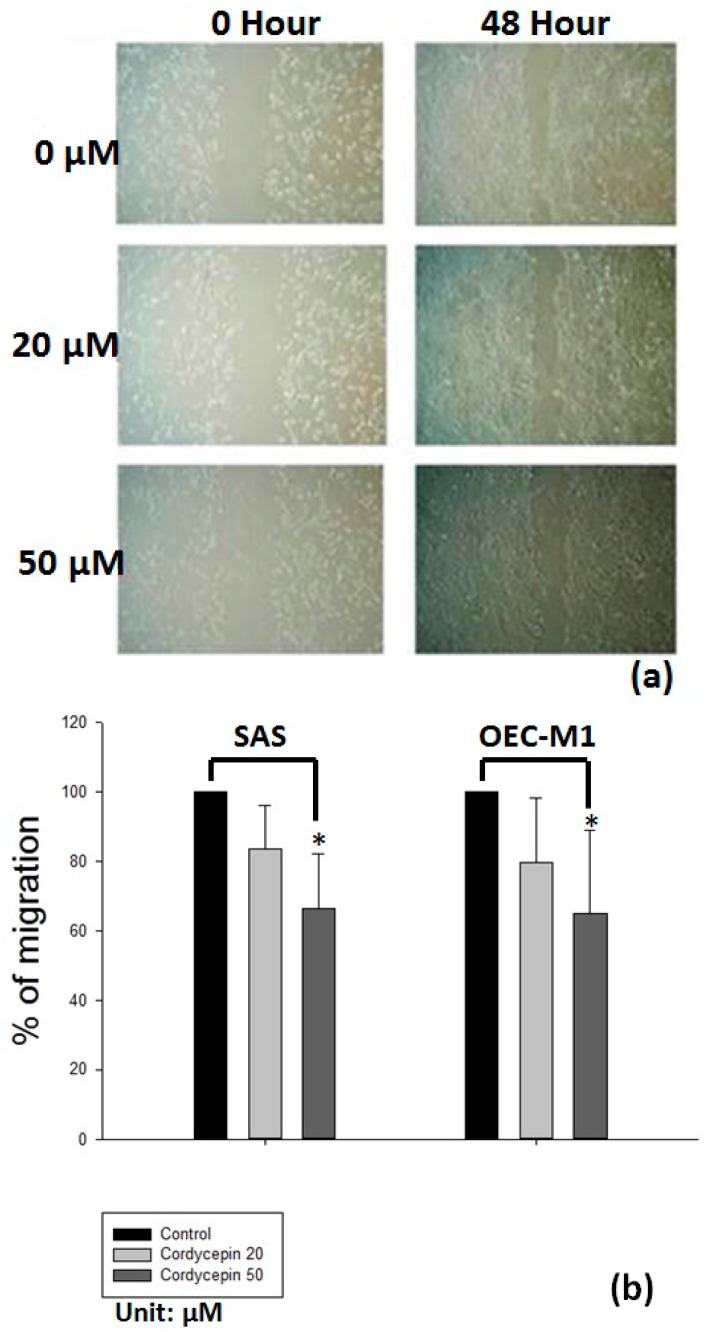
Cordycepin inhibited the migration ability of oral cancer cells. (**a**) Presents wound closure by SAS cells after 48 h of 0 μM, 20 μM and 50 μM cordycepin treatment. Codycepin at 50 μM significantly inhibited wound closure in both OSCC cells compared with the control or 20 μM cordycepin; (**b**) presents the mean values for delayed wound closure (three experiments) in SAS and OEC-M1 cells (* *p* < 0.05).

**Figure 7:**
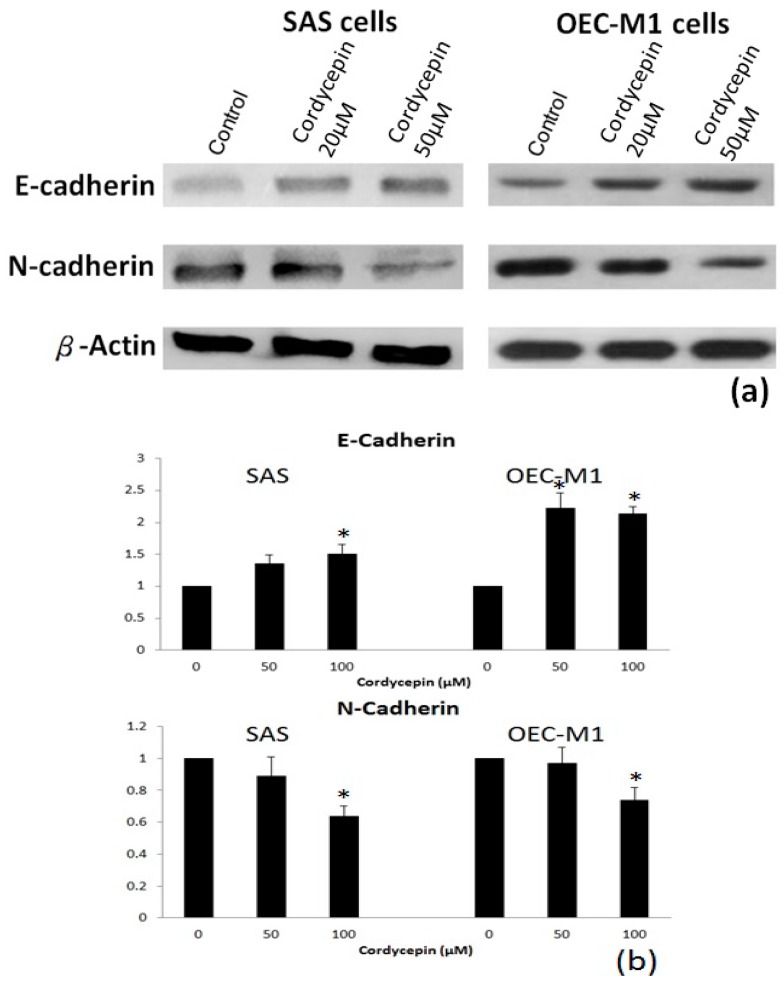
Cordycepin treatment inhibited the expression of epithelial-mesenchymal transition (EMT) markers. (**a**) Shows the results of immunoblotting of the epithelial (E-cadherin) and mesenchymal (*N*-cadherin) markers after 24 h of placebo or cordycepin treatment. Increased E-cadherin and decreased *N*-cadherin after cordycepin treatment at two different concentrations were observed; (**b**) demonstrates the results of statistical analysis of triplicate immunoblotting experiments (* *p* < 0.05).

The migration ability was prohibited in both HNSCC cancer cells after 48 h of treatment with cordycepin compared with the placebo group (Figure 6a,b). Furthermore, the migration area of SAS cells was reduced by 20 μM and 50 μM cordycepin to 82.6% and 63.5% (*p* < 0.05), respectively, compared with the placebo group. The migration area of OEC-M1 cells was also decreased by 20 μM and 50 μM cordycepin to 78.4% and 61.5% (*p* < 0.05), respectively. We then tested whether cordycepin could reverse EMT to inhibit the migration ability of OSCC cells. After treatment with 50 μM cordycepin, the expression of epithelial marker E-cadherin increased significantly (*p* < 0.05) compared with placebo treatment. Meanwhile, the expression of mesenchymal marker N-cadherin also decreased after treatment with 50 μM cordycepin (*p* < 0.05) (Figure 7a,b).

### 2.5. Validation of EMT Inhibition by Cordycepin in Xenograft Tumor

**Figure 8:**
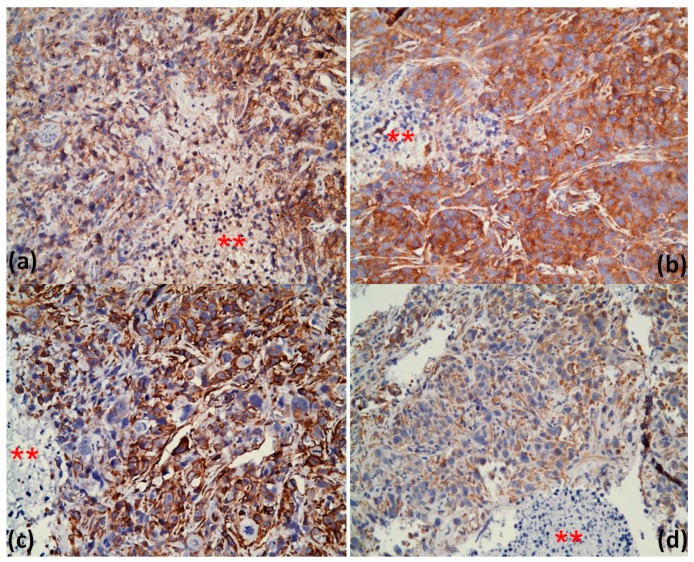
Cordycepin inhibited expression of EMT markers in xenograft tumors. Immunohistochemical staining was performed using antibodies against E-cadherin and vimentin. It showed that E-cadherin expression increased after cordycepin treatment (**a**: control group, **b**: 50 mg/kg cordycepin group). Vimentin staining intensity decreased after cordycepin treatment (**c**: control group, **d**: 50 mg/kg cordycepin group). The asterisk (**) represented areas of non-tumor part, which acted as an internal negative control for the IHC staining.

Immunohistochemical (IHC) staining of the xenograft tumors was performed with antibodies against E-cadherin and vimentin. We observed that the E-cadherin staining increased diffusely in cordycepin-treated tumors compared with the control group (Figure 8a,b). Furthermore, the staining intensity of vimentin decreased after cordycepin treatment (Figure 8c,d). The IHC staining results provided in vivo evidence that cordycepin delayed tumor growth accompanied by EMT inhibition. The alteration in the expression of *N*-cadherin in tumor after cordycepin treatment has similar, but less extent, trend to vimentin (data not shown).

## 3. Discussion

We demonstrated that cordycepin had growth inhibitory effect on the two OSCC cancer cell lines in a dose- and time-dependent manner. Cordycepin may have different growth inhibition effects on human normal counterpart cells in our study of human HFW fibroblasts and HaCat keratinocytes. We observed that cordycepin treatment resulted in OSCC cell apoptosis and increased caspase-3 activities. G2/M phase arrest increased, which was revealed by cell cycle analysis, in both cancer cells. However, the human HFW fibroblasts were not affected by cordycepin treatment. Two previous studies also focused on cordycepin in head and neck cancer [27,28]. These studies also revealed that cordycepin possessed apoptotic effect and arrested cell cycle at G2/M phase. Another study suggested that the underlying mechanisms may be related to c-Jun N-terminal kinase activation and associated p21WAF1 expression [24]. In several studies, cordycepin could slow the migration or invasion ability in various cancer types, mainly through the inhibition of metalloproteinase [25,29]. Our results showed that cordycepin restrained the migration in both the OSCC cell lines, which may result from EMT inhibition. Information available on the interaction between cordycepin and cancer cell EMT is insufficient. The upstream ZEB1, Twist1 and Snail transcription factors may be involved in the interaction [30,31]. Cancer cells with EMT features would behave more aggressively and possess significantly higher drug or radiation resistance [32,33]. Molecular EMT features also impart poor prognosis [34,35]. Compounds, such as cordycepin, which can inhibit EMT may be beneficial and may work synergistically with current cancer therapies. 

The unique metronomic treatment schedule has been researched exclusively in the field of chemotherapy. In our xenograft model, we used prolonged administration schedule (five days a week for eight consecutive weeks) in the tumor-bearing mice, which mimicked the metronomic chemotherapeutic drug delivery. Cordycepin has been tested in two previous xenograft animal studies. One used a single dose (maximal dose 25 mg/kg) of cordycepin injection in a melanoma model [26], and the other tested cordycepin with daily injections (10 mg/kg and 50 mg/kg) of 21 doses, but reported no evident toxicities [36]. Under our extended treatment duration, cordycepin demonstrated its ability to delay the OSCC tumor growth. Furthermore, the performance and functions of the organs were not compromised, indicating good tolerability of cordycepin metronomic treatment. Since cordycepin arrested the tumor cells at G2/M phase, it may be a potential radiosensitizer in increasing the effects of radiotherapy. The dose conversion from animal to human studies can be calculated and extrapolated using basal metabolic rate (BMR) relationship among species [37]. This combination would require further experimental validation.

Cordycepin is a naturally occurring compound and was used abundantly in the traditional Chinese medicine. Furthermore, it can be produced by fermentation and be easily synthesized. We found that cordycepin possesses anticancer effects and has minimal toxicity against tumor-bearing mice. All of the above properties may confer cordycepin the advantage of being integrated into clinical cancer therapies. However, cordycepin acts as a nucleotide analogue to inhibit cancer growth; therefore, the development of drug resistance could be further observed [38]. This issue should be addressed before it is used in cancer therapies.

## 4. Materials and Methods

### 4.1. Chemicals and Reagents

Cordycepin was obtained from Tokyo Chemical Industry (Kashiai, Japan). Mouse antibody against E-cadherin [39] and β-actin were purchased from Cell Signaling (Danvers, MA, USA), and rabbit anti-*N-*cadherin antibodies were from Millipore (Temecula, CA, USA) [40].

### 4.2. Cell Lines and Cell Culture—Oral Cancer and Normal Fibroblast

Two OSCC cell lines were used in this study. HFW cells (human fibroblasts) and HaCaT (human keratinocytes) were used as the normal counterparts to OSCC cell lines. The SAS cell line was purchased from ATCC (Manassas, VA, USA). OEC-M, HFW and HaCat cells were kindly provided by Professor CJ Liu (Taipei, Taiwan), TC Lee (Academica Sinica, Taipei Taiwan) and YY Chen (Taipei, Taiwan), respectively. SAS cells were maintained in DMEM medium supplemented with 10% *v/v* heat-inactivated fetal bovine serum (pH 7.4), 1% non-essential amino acid, 1% sodium pyruvate, and 1% streptomycin. OEC-M1 cells were cultured in Roswell Park Memorial Institute 1640 medium with 25 mM HEPES, 10% *v/v* heat-inactivated fetal bovine serum (pH 7.4) and 1% streptomycin. HFW cells were cultured with DMEM medium containing 10% *v/v* heat-inactivated fetal bovine serum (pH 7.4) and 1% streptomycin. HaCaT cells were maintained with DMEM medium containing 10% *v/v* heat-inactivated fetal bovine serum (pH 7.4), 1% glutamine and 1% streptomycin. All of the cells were incubated in a humidified atmosphere containing 95% air and 5% CO2 at 37 °C.

### 4.3. Animals and In Vivo Experiments

Male BALB/c nude mice, aged 6 to 8 weeks old, were bred and kept in accordance to the institutional ethical committee guidance (approval number : MMH-A-S-102-25). Tumor cells (100 μL, 1 × 10^6^) were injected subcutaneously into the right gluteal region of the nude mice. When an OSCC tumor grew to approximately 0.5 cm^3^, we randomly assigned the mice into 3 groups with similar weight and tumor size. Group A (*n* = 3) was the vehicle control group, which was treated with PBS intraperitoneally (IP). Group B and C (both *n* = 3) were treated with cordycepin at 25 mg/kg and 50 mg/kg IP, respectively. The mice were treated 5 days a week for 8 weeks or until mortality, whichever came first. The mice were sacrificed at 8 weeks considering animal welfare. Weight was recorded thrice a week and blood samples were collected weekly. We checked the white blood cell counts, serum ALT and creatinine levels. Tumors were dissected at the end of the study or at mortality. The tumor size was measured as (4π/3) × (width/2)^2^ × (length/2).

### 4.4. MTT Assay

The MTT assay was used to determine cell viability. In brief, cells were seeded in a 24-well plate with 2.5 × 10^4^ cells in 500 μL serum medium. Cells were treated with cordycepin at the indicated concentrations (0, 20, 50, 100, 200 μM). MTT (5 μL/mL) was added into each well to make up a total volume of 500 μL after 24, 48, 72 h of treatment and incubated for 4 h at 37 °C. Then, the medium was removed and 500 μL DMSO was added into each well for 30 min to dissolve the formazan crystal. The absorbance of each well was measured (570/630 nm) with an ELISA reader. 

### 4.5. Cell Cycle Analysis

Initially, SAS and OEC-M1 cells were treated with the indicated concentrations of cordycepin (0, 50, 100 μM) for 24 h. Then, the cells were collected, washed with PBS, and fixed in cold 70% ethanol. The cells were re-washed, re-suspended in cold PBS, and incubated with 10 mg/mL RNase and 1 mg/mL propidium iodide (PI) at 37 °C for 30 min in the dark. The samples were analyzed with flow cytometry BD FACSCalibur™ (BD Bioscience, San Jose, CA, USA). Percentage of the cancer cells in the G0/G1, S, and G2/M phases were determined with Cell Quest software (BD Bioscience, San Jose, CA, USA).

### 4.6. Liu’s Stain

The OSCC cells were seeded on 6-well plates and treated with indicated concentrations of the study compounds for 48 h. Then, the cells were washed with PBS twice and Liu’s stain solution A was added (45 s), followed by B (90 s). The cell morphology was observed and photographed under light microscope.

### 4.7. Caspase 3 Activity Assay

Caspase-3 activity was assessed by using the NucView 488 caspase-3 Substrate Kit (Biotium, Hayward, CA, USA, catalog No.10403). The oral cancer cells and HFW cells were treated with PBS, 50 μM and 100 μM cordycepin for 48 h. In brief, the treated cells were harvested and resuspended at a density of 1 × 10^6^ cells/mL in the buffer. The cell suspension (0.2 mL) was mixed with 1 μL of 1 mM substrate solution to obtain a final substrate concentration of 5 μM. Then, the mixture was incubated and protected from light at room temperature for 30 min. The caspase-3 activity was detected by flow cytometry (BD FACSCalibur™) and the data were analyzed by Cell Quest software 

### 4.8. Wound Closure Assay

The wound closure assay was performed to examine the migration ability of the oral cancer cells. In brief, the SAS and OEC-M1 cells were grown to full confluency in the silicone inserts (Grid-500, ibid GmbH, City, Germany) with a defined 500 μm cell-free gap and incubated with or without cordycepin (0, 20, and 50 μM). The wound gap was observed by phase microscopy and photographed 48 h after treatment. The migration area was measure and quantified using software image J (Bethesda, MD, USA).

### 4.9. Protein Extraction and Western Blot Analysis

Whole-cell lysates were prepared from cells treated with cordycepin for 24 h. The membrane was blocked with 5% skim milk and immunoblotted with primary antibodies against E-cadherin, N-cadherin, β-actin at 4 °C overnight. This was followed by the addition of horseradish peroxidase-labeled secondary antibodies (Chemicon, Single Oak Drive, Temecula, CA, USA) and development using the enhanced chemiluminescence system (Amersham Pharmacia, Piscataway, NJ, USA). The expression of β-actin was used as an internal control. The protein bands were measured and quantified using software image J.

### 4.10. Immunohistochemistry Staining of E-Cadherin and Vimentin in SAS OSCC Xenograft

For IHC analyses, excised tumors were fixed in formalin and embedded in paraffin. All tumor samples were 4 μm thick tissue sections. These sections were treated with heat-induced antigen retrieval at 95 °C in sodium citrate buffer (10 mM, pH 6.0) for 30 min. Endogenous peroxidase was blocked by 3% hydrogen peroxide (H_2_O_2_) at room temperature for 30 min. Nonspecific reaction were blocked by BlockPRO™ Blocking Buffer (Visual Protein, Taipei, Taiwan) at room temperature for 30 min. Sections were then incubated with primary antibody against E-Cadherin (1: 250; GeneTex, Irvine, CA USA) and vimentin (1: 50; Cell Signaling, Denver, MA, USA) overnight at 4 °C, followed by using VECTASTAIN^®^ ABC Kit (Rabbit IgG)( PK-6101; Vector Laboratories, Peterborough, UK). Sections were stained with Liquid DAB+ Substrate Chromogen System (DAKO, Carpinteria, CA, USA) and counterstained with hematoxylin. An oncology pathologist expert helped in examining the stained slides and estimating the expression levels of E-cadherin and vimentin. The IHC staining was then observed and photographed under light microscope.

### 4.11. Statistics

All experiments were performed and repeated at least thrice. Data were analyzed from 3 independent experiments and all the values were expressed mean ± standard deviation. The statistical analysis of data was performed with IBM SPSS Statistics 22 (IBM Co., New York, NC, USA). One-way analysis of variance (ANOVA) method was used to determine the statistical significance among groups. A *p* value of < 0.05 was considered statistically significant.

## 5. Conclusions

In conclusion, metronomic administration of cordycepin showed effective tumor control, prolonged survival and low toxicities accompanied by inhibition of EMT in OSCC cells. Further investigation is warranted to assess the clinical therapeutic potential of cordycepin.
